# Surveillance of catheter-related infections: the supplementary role of the microbiology laboratory

**DOI:** 10.1186/s12879-014-0743-5

**Published:** 2015-01-08

**Authors:** Wilhelmina Strasheim, Martha M Kock, Veronica Ueckermann, Ebrahim Hoosien, Andries W Dreyer, Marthie M Ehlers

**Affiliations:** Department of Medical Microbiology, University of Pretoria, Pretoria, South Africa; National Health Laboratory Service, Tshwane Academic Division, Pretoria, South Africa; Department of Internal Medicine, University of Pretoria, Pretoria, South Africa; Centre for Tuberculosis, National Institute of Communicable Diseases, Johannesburg, South Africa

**Keywords:** Healthcare-associated infections, Catheter-related infections, Catheter-related bloodstream infections, Central line-associated bloodstream infections, South Africa

## Abstract

**Background:**

The burden of catheter-related infections (CRIs) in developing countries is severe. In South Africa, a standardised surveillance definition does not exist and the collection of catheter days is challenging. The aim of the study was to provide baseline data on the prevalence of CRIs and to describe the epidemiology of CRI events within a tertiary academic hospital.

**Methods:**

Surveillance was laboratory-based and conducted for a six month period. A microbiologically confirmed CRBSI (MC-CRBSI) event was defined as the isolation of the same microorganism from the catheter and concomitant blood cultures (BCs), within 48 h of catheter removal, which were not related to an infection at another site.

**Results:**

A total of 508 catheters, removed from 332 patients, were processed by the laboratory, of which only 50% (253/508 removed from 143/332 patients) of the catheters were accompanied by BCs within 48 h. Sixty-five episodes of MC-CRBSI in 57 patients were detected, involving 71 catheters and 195 microbial isolates. The institutional prevalence rate was 3.7 episodes per 1 000 admissions and 5.8 episodes per 10 000 in-patient days. Catheter day data was collected in only six wards of the hospital. The pooled laboratory incidence was 10.1 MC-CRBSI episodes per 1 000 catheter days, whereas the hospital-based central line-associated bloodstream infection (CLABSI) rate was pooled at 5.7 episodes per 1 000 catheter days. The majority of patients had an underlying gastro-intestinal condition (33%; 19/56) with a non-tunnelled, triple-lumen central venous catheter, placed in the subclavian vein (38%; 27/71). The most predominant pathogen was methicillin-resistant *Staphylococcus epidermidis* (28%; 55/195), followed by extensively-drug resistant *Acinetobacter baumannii* (18%; 35/195).

**Conclusions:**

Catheter-related infection prevention and control efforts require urgent attention, not only to keep patients safe from preventable harm, but to prevent the spread of multidrug resistant microorganisms.

## Background

Catheter-related infections [CRI(s)] in sub-Saharan Africa pose a significant threat to a hospitalized patient’s safety [1,2]. Not only are CRIs associated with increased mortality, these infections contribute an increased length of hospital stay and increased healthcare costs, which is problematic in a limited-resource setting, such as South Africa [3,4].

A focus on education and strict adherence to infection prevention and control programmes are associated with a reduction in CRI rates [5]. However, the first step in reducing CRIs, prior to the implementation of education and prevention programmes, is to define the extent of the problem through surveillance [4,6]. Effective surveillance measures are dependent on the adoption of a standardised case definition [7]. However, there is lack of uniformity of the case definition of a CRI among different organisations in various geographical locations [8-11].

In South Africa, tuberculosis, HIV/AIDS, maternal and child health are national priorities and the improvement of patient safety and healthcare quality has only recently been identified as important [12]. Surveillance of healthcare-associated infections and the accompanying definitions are therefore not yet established in South Africa [13]. In 2009, the “Best Care…Always!” (BCA) initiative was launched to increase the implementation speed of quality improvements throughout healthcare facilities and the campaign adopted a simplified version of the United States of America’s Centers of Disease Control and Prevention’s National Healthcare Safety Network’s (CDC/NHSN’s) definition [14].

However, the CDC/NHSN’s definition might not be ideal for the situation in South Africa, since it requires extensive education and training, is laborious and subjective [15]. In addition, South Africa is facing a national shortage of nursing staff and the collection of denominator data (i.e. the number of catheter days) is a huge challenge [3,16,17]. A simplified laboratory-based definition might be an alternative surveillance measure as suggested by Rodríguez-Créixems and colleagues, since the data is readily available and can be reported against a denominator of either patient admissions or in-patient days [18].

The aim of the study was to provide baseline data on CRIs from laboratory records and to describe patient, catheter and pathogen characteristics of CRI events within a tertiary academic hospital in South Africa. This is the first report, to the extent of the authors’ knowledge, to describe CRIs in such detail in an institution in sub-Saharan Africa.

## Methods

### Study setting

The observational study was laboratory-based. Only intravascular catheters submitted with concomitant blood culture(s) [BC(s)] received from a tertiary academic hospital, during May to October 2013, were included. The 832-bed hospital acts as a referral hospital in the region; providing specialised services to the public.

### Data collection and definitions

Laboratory surveillance was conducted in accordance with the Infectious Diseases Society of America’s and the Irish’s national CRI committee’s definitions [11,19]. Briefly, a microbiologically confirmed catheter-related bloodstream infection (MC-CRBSI) was defined as a bloodstream infection occurring within 48 h of catheter removal and the subsequent isolation of the same microorganism from the catheter culture as on the BC(s) [11]. Additional criteria were added to include events caused by common commensals, such as *Staphylococcus epidermidis*, in which only a single BC bottle flagged positive [19]. The genetic relationship between catheter tip and BC isolates of common commensals were confirmed with pulsed-field gel electrophoresis. Catheter tip and BC isolates were considered identical, if the pulsotype’s banding patterns showed ≥ 80% similarity (Applied Maths, Kortrijk, Belgium). The event was excluded if the similarity values were < 80% (Results not shown).

Patient and catheter details were collected retrospectively. Patient details included: i) underlying clinical condition, ii) length of hospitalisation up and to the MC-CRBSI event, iii) length of catheterisation (number of days elapsed from catheter insertion to catheter removal), iv) antimicrobial exposure, v) chronic illness (HIV, diabetes, renal failure) and vi) other risk factors (malnutrition, loss of skin integrity, neutropenia, total parental nutrition administration) associated with CRIs. Catheter details included: i) type of vessel occupied, ii) insertion site, iii) physical length of the catheter (long or short) and iv) any special characteristics associated with the catheter, such as the presence of cuffs, antimicrobial impregnation and the number of lumens.

The aetiological agent(s) of an event and it’s accompanied antimicrobial susceptibility pattern were also recorded. Multidrug-resistance (MDR), extensively drug-resistance (XDR) and pandrug-resistance (PDR) were defined for the six most prevalent bacteria according to international standards [20].

### Laboratory procedures

Blood cultures were processed using the BacT/ALERT 3D system (bioMérieux, France). Quantitative BCs are not performed routinely in the microbiology laboratory and MC-CRBSI diagnosed by differential time to positivity was excluded, due to labelling issues. Catheters were cultured according to a semi-quantitative method [21]. The growth of ≥15 colony forming units (cfu) was regarded as significant. All positive catheter and BCs were further managed according to standard microbiological procedures. Identification and antimicrobial susceptibility testing of microbial isolates were performed with the VITEK® 2 system (bioMérieux, France). Isolates showing intermediate susceptibility were considered resistant.

### Statistical analysis

Data recording and statistical analysis were performed using Excel (Microsoft, Redmond, Washington, USA). Categorical variables were reported as numbers and percentages, whereas continuous variables were reported as means and standard deviations. The incidence (i.e. the number of new cases divided by the population at risk, which is only catheterised patients) of MC-CRBSI was expressed as the total number of episodes per 1 000 catheter days and was calculated by dividing the total number of MC-CRBSI episodes with the total number of catheter days, multiplied by a 1 000. The prevalence (i.e. the number of cases divided by the population) of MC-CRBSI was expressed as the total number of episodes per 1 000 patient admissions, as well as per 10 000 in-patients days and was calculated by dividing the total number of MC-CRBSI episodes with the total number of patients admitted or the total number of in-patient days, multiplied by 1 000 or 10 000, respectively.

### Ethical approval

Ethical approval was obtained from the Faculty of Health Sciences Research Ethics Committee of the University of Pretoria (Protocol number: 118/2013). Informed consent was waivered, since the study was observational and patient care was not influenced.

## Results

### Episodes of MC-CRBSI

A total of 508 catheter tips removed from 332 patients were processed by the microbiology laboratory during the six month study period. Only 50% of the catheters (253/508 removed from 143/332 patients) were accompanied by BCs within 48 h. A total of 65 episodes of MC-CRBSI in 57 patients were identified. The overall prevalence for the institution was calculated at 3.7 episodes per 1 000 admissions (65 episodes ÷ 17 559 admissions × 1 000) and 5.8 per 10 000 in-patient days (65 episodes ÷ 112 452 in-patient days × 10 000).

The number of catheter days was only available for six wards in the hospital, which is currently under BCA surveillance. The MC-CRBSI pooled incidence density was calculated at 10.1 episodes per 1 000 catheter days for these six wards (44 episodes ÷ 4 373 catheter days × 1 000). The reported CLABSI rate by the BCA campaign for the corresponding period was pooled at 5.7 episodes per 1 000 catheter days (25 episodes ÷ 4 373 catheter days × 1 000). The relationship between the number of catheters submitted for culture; the number of catheters accompanied by BCs within 48 h; the BCA campaign’s CLABSI rate and the laboratory MC-CRBSI rate are shown in Table 1.

**Table 1:** **Comparision between hospital-based CLABSI rates and laboratory-based MC-CRBSI rates**

**Ward**	**A**	**B**	**C**	**D**	**E**	**F**	**Pooled**
**Hospital-based surveillance**							
Number of CLABSI* events	0	5	5	7	7	1	25
CLABSI incidence per 1 000 catheter days	0	5.6	4.9	7.7	11.3	6.5	5.7
**Laboratory-based surveillance**							
Number of catheters submitted for culture	85	49	88	70	19	8	319
Number of catheters accompanied by BCs	44	10	77	52	6	8	197
Number of MC-CRBSI events	10	2	13	12	3	4	44
MC-CRBSI incidence per 1 000 catheter days	12.9	2.2	12.7	13.2	4.9	25.8	10.1
MC-CRBSI prevalence per 1 000 admissions	30.1	11.2	65.6	122.5	19.6	22	38.5
**Denominators** ^**#**^							
Number of central line days	776	891	1023	912	617	155	4374
Number of patient admissions	332	179	198	98	153	182	1142

A = High care, multidisciplinary ward; B = Neurosurgery ICU; C = Trauma and Surgery ICU; D = Medical and Pulmonology ICU; E = Cardiothoracic ICU; F = Paediatric Medical ICU.

CLABSI = central line-associated bloodstream infection; BCs = blood cultures; MC-CRBSI = microbiologically confirmed catheter-related bloodstream infection.

*CLABSI definition from Best Care…Always! = Occurrence of a primary bloodstream infection in a patient with a central line *in situ* or where infection occurs within 48 h of the removal of the central line, where no other possible source of the bloodstream infection could be identified.

^**#**^The number of in-patient days was only available for the hospital as a whole and could therefore not be broken down per ward.

Thirty-two percent (32.3%; 21/65) of MC-CRBSI episodes were detected in non-surveillance wards, which included: i) paediatric surgery (38.1%; 8/21), ii) general surgery (14.3%; 3/21), iii) oncology (6.5%; 2/21), iv) nephrology/peritoneal (6.5%; 2/21), v) neonatal intensive care unit (ICU) (6.5%; 2/21), vi) vascular surgery (4.8%; 1/21), vii) neurosurgical (4.8%; 1/21), viii) urology and gynaecology (4.8%; 1/21) and ix) plastic/maxillofacial/ophthalmology (4.5%; 1/21).

### Clinical details of the patients

The clinical characteristics of the patients are summarised in Table 2. Six patients had multiple MC-CRBSI episodes. Each of these episodes occurred in the same location as the first event, but a different microorganism was responsible and occurred within 18.9 days (standard deviation (SD) = ± 11 days) of another. Six patients were HIV-positive, two patients were exposed to HIV and the HIV-status was unknown for two patients. Eighty-nine percent (89.5%; 51/57) of patients had been exposed to one or more antimicrobial agent (Table 3). A total of 31% (40/131) of antimicrobial exposures were with last-resort antimicrobial agents (e.g. glycopeptides, linezolid, tigecycline and colistin).

**Table 2:** **Patient demographics, underlying conditions, chronic illnesses and risk factors for MC-CRBSI events**

	**Adults % (n = 40)**	**Children % (n = 5)**	**Infants % (n = 12)**
**Number of MC-CRBSI episodes per patient**			
1	87.5 (35)	80.0 (4)	100.0 (12)
2	10.0 (4)	-	-
3	2.5 (1)	20.0 (1)	-
**Demographics**			
Mean age (±SD)	44 years (±14.9)	4 years (±3.16)	31 days (±21.4)
% Male	42.5 (17)	100.0 (5)	66.7 (8)
**Underlying conditions** ^**^**^			
Gastro-intestinal	27.5 (11)	20.0 (1)	58.3 (7)
Nephrology	22.5 (9)	-	-
Trauma	10.0 (4)	-	8.3 (1)
Dermatology (includes burned patients)	7.5 (3)	40.0 (2)	-
Surgery (amputation)	7.5 (3)	-	-
Respiratory	5.0 (2)	-	25.0 (3)
Malignancy	5.0 (2)	20.0 (1)	8.3 (1)
Neurologic	5.0 (2)	-	-
Cardiovascular	2.5 (1)	20.0 (1)	-
Endocrinology	2.5 (1)	-	-
Gynaecology	2.5 (1)	-	-
Unknown	2.5 (1)	-	-
**Days of hospitalisation** ^**#**^ **(mean) (±SD)**	29.0 (±22.2)	30.0 (±19.3)	27.1 (±14.9)
Unknown per number of events % (n)	13.3 (6/45)	14.3 (1/7)	16.6 (2/12)
**Chronic illness** ^**^**^			
None	57.5 (23)	80.0 (4)	83.3 (10)
Renal failure	22.5 (9)	-	-
HIV-positive	12.5 (5)	20.0 (1)	(2 exposed)
Diabetes	12.5 (5)	-	-
Unknown	2.5 (1)	-	-
**Risk factors** ^**^**^			
None	40.0 (16)	-	50.0 (6)
TPN administration	42.5 (17)	40.0 (2)	33.3 (4)
Malnutrition	12.5 (5)	20.0 (1)	-
Loss of skin integrity	7.5 (3)	40.0 (2)	8.3 (1)
Neutropenia	2.5 (1)	20.0 (1)	8.3 (1)
Unknown	2.5 (1)	-	-
**Length of catheterisation**	11.1 (±5.5)	15.7 (±6.1)	11.9 (±2.5)
Unknown per number of catheters % (n)	21.6 (11/51)	14.3 (1/7)	33.3 (4/12)

SD = standard deviation; TPN = total parenteral nutrition.

^^^Incomplete clinical details for a single patient, six patients (five adults and one child) had more than two risk factors present at once, which will explain why percentages do not add up to 100%.

^#^the mean length of hospitalisation was calculated for each MC-CRBSI event in patients with multiple events.

**Table 3:** **Antimicrobial exposure of patients with MC-CRBSI events**

**Number of antimicrobials a patient had been exposed to % (n = 57)**	
None	5.3 (3)
1	21.1 (12)
2	33.3 (19)
3	15.8 (9)
4	8.8 (5)
5	7.0 (4)
6	3.5 (2)
Unknown	5.3 (3)
**Antimicrobial agents, according to class, to which patients were exposed to** ^**#**^ **% (n = 131 exposure events)**	
Carbapenems	29.8 (39)
Glycopeptides	15.3 (20)
Polymyxins (colistin)	10.7 (14)
Antifungals	9.9 (13)
β-lactamase inhibitors (piperacillin/tazobactam)	6.9 (9)
Penicillin	4.6 (6)
Folate pathway inhibitors	3.8 (5)
Oxazolidnones (linezolid)	3.8 (5)
Aminoglycosides	3.1 (4)
Extended-spectrum cephalosporins	3.1 (4)
Non extended-spectrum cephalosporins	3.1 (4)
Rifampicin	2.3 (3)
β-lactamase inhibitors (amoxicillin/clavulanic acid)	1.5 (2)
Rifafour	0.8 (1)
Macrolides	0.8 (1)
Glycylcyclines (tigecycline)	0.8 (1)

^#^Carbapenems = ertapenem, meropenem and imipenem; glycopeptides = teicoplanin and vancomycin; antifungals = fluconazole, amphotericin B and voriconazole; folate pathway inhibitors = bactrim (sulfamethoxazole and trimethoprim) and dapsone; extended-spectrum cephalosporins = ceftriaxone (rocephin); non extended-spectrum cephalosporins = prophylactic cefazolin (kefzol); macrolides = clarithromycin.

### Characteristics of the catheters

A total of 71 catheters, accompanied by 112 positive BCs, were implicated in the 65 MC-CRBSI episodes. The number of BCs submitted per episode and the characteristics of the catheters are summarised in Table 4. A total of 38% (27/71) of the catheters were a non-tunnelled, triple lumen, central venous catheter, inserted in the subclavian vein. All catheters were non-cuffed and not impregnated with antimicrobials.

**Table 4:** **Characteristics of catheters that acted as the source of the MC-CRBSI episodes**

**Number of catheters per MC-CRBSI episode % (n = 71)**						
1	91.5 (65)					
2	8.5 (6)					
**Type of catheter combinations % (n = 6)**						
CVP and arterial	83.6 (5)					
CVP and VasCath	16.7 (1)					
**Number of concomitant BCs submitted % (n = 65 episodes)**						
1	23.1 (15)					
2	46.2 (30)					
3	3.1 (2)					
≥4	27.7 (18)					
**Number of positive concomitant BCs % (n =65 episodes)**						
1	47.7 (31)					
2	36.9 (24)					
3	10.8 (7)					
≥4	4.6 (3)					
**Type of catheter**	**CVP**	**Arterial**	**VasCath**	**Broviac**	**Peripheral venous**	**Umbilical**
	**% (n = 51)**	**% (n = 7)**	**% (n = 7)**	**% (n = 4)**	**% (n = 1)**	**% (n = 1)**
**Insertion site**						
Subclavian	66.7 (34)		-	75 (3)	-	-
Peripheral	-	28.6 (2)	-	-	100 (1)	-
Radial	-	71.4 (5)	-	-	-	-
Femoral	3.9 (2)	-	14.3 (1)	-	-	-
Internal jugular	21.6 (11)	-	71.4 (5)	-	-	-
Umbilical	-	-	-	-	-	100 (1)
Unknown	7.8 (4)	-	14.3 (1)	25 (1)	-	-
**Physical length of the catheter**						
Short	94.1 (48)	100 (7)	100 (7)	100 (4)	100 (1)	100 (1)
Long	3.9 (2)	-	-	-	-	-
Unknown	1.9 (1)					
**Type of vessel occupied**						
Central	100 (51)	-	100 (7)	100 (4)	-	-
Arterial	-	100 (7)	-	-	-	-
Peripheral venous	-	-	-	-	100 (1)	100 (1)
**Insertion site pathway to the vessel**						
Non-tunnelled	88.2 (45)	100 (7)	85.7 (6)	100 (4)	100 (1)	100 (1)
Tunnelled	9.8 (5)	-	14.3 (1)	-	-	-
Unknown	1.9 (1)		-	-	-	-
**Number of lumens**						
1	-	100 (7)	-	25 (1)	100 (1)	-
2	9.8 (5)	-	28.6 (2)	25 (1)	-	100 (1)
3	84.3 (43)	-	71.4 (5)	25 (1)	-	-
Unknown	5.8 (3)	-	-	25 (1)	-	-
**Length of catheterisation**						
% Unknown	17.6 (9)	71.4 (5)	14.3 (1)	0	0	100
Number of days (mean ± SD)	12.0 (5.4)	8.5 (2.1)	11.5 (5.5)	11 (6.8)	9 (n/a)	-

BCs = blood culture(s); CVP = central venous catheter; SD = standard deviation.

### Microbiology and susceptibility patterns

A total of 195 microorganisms were isolated in the 65 MC-CRBSI episodes. Five MC-CRBSI episodes were polymicrobial. The combinations of the polymicrobial infections were as follow: i) *S. epidermidis* and *Achromobacter* species, ii) *Klebsiella pneumoniae* and *Enterobacter cloacae*, iii) *S. aureus* and *Enterococcus faecalis*, iv) *S. epidermidis* and *Acinetobacter baumannii* complex and v) *Pseudomonas aeruginosa* and *K. oxytoca*. Forty-nine percent (48.5%; 34/70) of the MC-CRBSI episodes were caused by Gram-negative bacteria; 45.7% (32/70) by Gram-positive bacteria and 5.7% (4/70) by *Candida* species (Table 5). Complete antimicrobial susceptibility patterns are described in Table 6 for the most prevalent bacteria implicated in MC-CRBSI episodes. All *Enterococcus* isolates were sensitive to vancomycin. *Proteus mirabilis* and *Escherichia coli* isolates were all extended-spectrum β-lactamase producers.

**Table 5:** **Distribution of microorganisms implicated in MC-CRBSI episodes**

**Aetiological agent**	**% (n = number of isolates)**	% (n = number of MC-CRBSI events)*	**Rank based on number of events**
**Gram-positive bacteria**			
Staphylococci			
*S. epidermidis*	28.2 (55)	25.7 (18)	1
*S. aureus*	8.2 (16)	10 (7)	3
*S. haemolyticus*	4.1 (8)	4.29 (3)	5
Enterococci			
*E. faecalis*	4.1 (8)	4.29 (3)	5
*E. faecium*	1.0 (2)	1.43 (1)	6
**Gram-negative bacteria**			
Environmental gram-negatives			
*Acinetobacter baumannii* complex	17.9 (35)	20 (14)	2
*Pseudomonas aeruginosa*	7.2 (14)	7.14 (5)	4
*Stenotrophomonas maltophilia*	1.5 (3)	1.43 (1)	6
*Achromobacter* species	1.0 (2)	1.43 (1)	6
*Enterobacteriaceae*			
*Enterobacter cloacae*	8.7 (17)	7.14 (5)	4
*Klebsiella pneumoniae*	6.2 (12)	7.14 (5)	4
*Klebsiella oxytoca*	2.6 (5)	1.43 (1)	6
*Escherichia coli*	2.6 (5)	1.43 (1)	6
*Proteus mirabilis*	1.5 (3)	1.43 (1)	6
**Fungi**			
*Candida* species			
*C. albicans*	3.6 (7)	4.29 (3)	5
*C. parapsilosis*	1.5 (3)	1.43 (1)	6

*Five MC-CRBSI events were polymicrobial, which will explain why the total number of MC-CRBSI-events is divided by 70.

**Table 6:** **Antimicrobial susceptibility profiles of microorganisms implicated in MC-CRBSI episodes**

**Antimicrobial susceptibility profile according to antimicrobial category**	**% isolates resistant**					
	***S. epidermidis*** **(n = 55)**	***S. aureus*** **(n = 16)**	***A. baumannii*** **complex (n = 35)**	***E. cloacae*** **(n = 17)**	***P. aeruginosa*** **(n = 14)**	***K. pneumoniae*** **(n = 12)**
Anti-staphylococcal β-lactams (oxacillin)	96	75	-	-	-	-
Penicillin (ampicillin)	-	-	-	-	-	100
β-lactamase inhibitors (amoxicillin/clavulanic acid)	-	-	100	-	-	33
β-lactamase inhibitors (piperacillin/tazobactam)	-	-	-	53	36	33
Cephamycins (cefoxitin)	-	-	-	-	-	17
Non-ES cephalosporins (cefuroxime)	-	-	-	71	-	33
ES cephalosporins (cefotaxime^#^, ceftazidime, cefepime)	-	-	100	53	21	33
Carbapenems (ertapenem^#^, meropenem, imipenem)	-	-	100	18	36	0
Aminoglycosides (amikacin^*^, gentamicin)	80	63	86	0	14	17
Fluoroquinolones (ciprofloxacin, moxifloxacin^^^)	55	75	49	0	14	17
Macrolides (erythromycin)	84	75	-	-	-	-
Linosamides (clindamycin)	76	63	-	-	-	-
Linezolid	0	0	-	-	-	-
Glycopeptides (teicoplanin, vancomycin)	0	0	-	-	-	-
Tetracycline	93	63	-	-	-	-
Glycylcycline (tigecycline)	0	0	3	12	-	17
Fusidic acid	24	0	-	-	-	-
Rifampicin	47	19	-	-	-	-
Polymyxins (colistin)	-	-	0	6	14	0
Folate pathway inhibitors (trimethoprim/ sulphamethoxazole)	78	75	-	12	-	17
**Acquired resistance profiles**						
S	11	25	0	47	79	67
MDR	89	75	0	53	7	33
XDR	0	0	100	0	0	0
PDR	0	0	0	0	14	0

ES = extended-spectrum; S = sensitive.

MDR = multidrug-resistant, non-susceptible to ≥1 agent in ≥ 3 antimicrobial categories.

XDR = extensively drug-resistant, non-susceptible to ≥ 1 agent in all but ≤ 2 antimicrobial categories.

PDR = pandrug-resistant, non-susceptible to all antimicrobial agents tested [20].

- = not applicable to the specific bacteria, ^#^not tested (n/t) for in *P. aeruginosa,* *n/t for in Gram-positive bacteria*,* ^^^n/t for in Gram-negative bacteria.

## Discussion

Catheter-related infections pose an unrecognised threat to patient safety. The results of the study showed that laboratory surveillance plays an important role in establishing baseline data of CRIs in a setting with limited resources. The number of patient admissions or the number of in-patient days can effectively describe the hospital-wide prevalence of these infections, especially where the collection of catheter days is problematic, as previously showed by Rodríguez-Créixems and colleagues [18].

Comparing MC-CRBSI rates between surveillance networks are difficult, since the numerator (i.e. case definition of a CRI) and the denominators (i.e. 1 000 catheter days, in-patient days or admissions) applied, vary greatly. If one compare our MC-CRBSI rate (10.1 per 1 000 catheter days; 0.57 per 1 000 in-patient days or 3.7 per 1 000 admissions), regardless of the reporting denominator, to studies in Europe (2.1 per 1 000 catheter days) [19], Spain (2.5 per 1 000 admissions) [18], Israel (0.21 per 1 000 in-patient days) [22] and by the International Nosocomial Infection Control Consortium (4.8 per 1 000 catheter days) [23], our MC-CRBSI rate was higher.

Reasons argued to contribute to a higher CRI rate includes: i) a low nurse-to-patient ratio, ii) a lack of education, iii) nurse inexperience and iv) a lack of infection control legislation [4]. In South Africa, a survey of public medical and surgical unit nurses showed that the nurse-to-patient ratio ranged from 8.75 to 32 [17], whereas a national audit showed that there are 0.3 trained ICU nurses per ICU/high care bed [24]. Furthermore, only 25.6% (1 490/5 821) of ICU nurses were formally trained in critical care and 42.8% (1 961/4 578) had zero to five years of experience [24]. Draft legislation passed in 2008 by the South African National Department of Health, proposed one infection control practitioner per 200 beds [13]. A human resource capacity survey followed and found that no healthcare facility had the required number of infection control practitioners and that 45.8% (116/253) of the respondents were not formally employed; performed infection control activities part-time or out of personal interest and did not have additional support from an epidemiologist or a microbiologist [13].

Studies comparing the laboratory-based MC-CRBSI rates with the classical hospital-based CLABSI rates are scarce [18]. If one compares our laboratory-based MC-CRBSI rate to the BCA campaign’s CLABSI rate, one will note that the MC-CRBSI rate was higher in most of the wards. This seems odd at first, since hospital-based CLABSI surveillance tends to overestimate the actual CRI rate, whereas laboratory-based MC-CRBSI tends to underestimate the actual CRI rate [25]. However, an important contributing factor, which will explain the difference between the two rates, is the point at which the CRI incidence is measured. Laboratory-based MC-CRBSI rates are measured at the point of catheter removal, whereas hospital-based CLABSI rates are measured at the point of catheter insertion. Comparison of CLABSI rates with MC-CRBSI rates may in future be used as a measurement to evaluate the effectiveness of catheter maintenance practices against catheter insertion practices. The two wards with a higher CLABSI rate and a lower MC-CRBSI rate had a low number of catheter tips accompanied by BCs within 48 h (Table 1) and microbiological documentation, as such, is known to bias the true CRI rate [26].

*Staphylococcus epidermidis* was the most prevalent aetiological agent, followed by *Acinetobacter baumannii* complex. It can be argued that the criteria to include a single positive BC for common commensals can inflate the prevalence of *S. epidermidis*. However, the genetic relationship between the catheter tip and BC isolates of *S. epidermidis* isolates was confirmed with pulsed-field gel electrophoresis. A notable exception to the distribution of CRI pathogens in the study is the predominance of *A. baumannii*. Enterococci and *Candida* species are more predominant in the USA, whereas *Pseudomonas aeruginosa* and *Escherichia coli* are more predominant in Europe [27,28]. The high proportion of the MC-CRBSI episodes caused by Gram-negative bacilli is suggestive of a potential bias either at institutional level or on patient-level. A study by Bouza and colleagues showed that patients with a Gram-negative CRI were more likely to have an underlying gastro-intestinal condition and were previously exposed to antimicrobials, which were similar to the patients involved in the current study [29].

A shift in the predominance of CRI pathogens, from Gram-positive to Gram-negative bacteria, is widely reported, which suggests that central line insertion bundles are more effective in reducing Gram-positive CRI than Gram-negative CRI [18,22,30-32]. The high prevalence of *S. epidermidis* CRI therefore implies failure towards central line insertion bundle adherence. Urgent attention needs to be paid to the education and training of healthcare personnel involved in catheter insertion, not only to decrease the rate of MC-CRBSI, but to ensure patient safety.

*Acinetobacter baumannii* is listed by the Infectious Disease Society of America as one of the six top-priority dangerous microorganisms, which makes the predominance of this pathogen in the study a matter of concern. The hospital environment (i.e. bed rails, mattresses, medical equipment, colonised or infected patients and the hands of healthcare workers) is an ecological niche for *A. baumannii* and resistance to desiccation, disinfections and antimicrobials makes eradication of this pathogen difficult [33]. All *A. baumannii* isolates was defined XDR in the study, with isolates only being susceptible to colistin. Although empirical antibiotic therapy for Gram-negative CRIs is based on local epidemiology, caution should be exercised in recommending colistin monotherapy in the management of CRI patients. Colistin-resistance in *A. baumannii,* which is associated with the inappropriate use thereof, can emerge rapidly, due to chromosomal mutations [34]. Combination therapy of colistin and a carbapenem should rather be used to prevent the emergence of colistin-resistant *A. baumannii* [34]. Extensive environmental decontamination, such as daily chlorhexidine bathing and oral care of colonised or infected patients, as well the use of an oxidizing disinfectant (Virkon S; potassium peroxomonosulphate 50%, sodium alkyl benzene sulphonate 15% and sulphamic acid 5%), should be considered to control and prevent further spread of *A. baumannii* [35,36].

The study had a number of limitations. First, it is important to note that the definition applied is highly specific and that some cases of CRIs might have been missed. In future, criteria, such as the clinical improvement of the patient after line removal, which is suggestive of a CRI and other diagnostic methods, such as differential time to positivity, which might be more appropriate in oncology and haematological patient populations, should be included to obtain a more reliable CRI rate. Second, the Acute Physiological Assessment and Chronic Health Evaluation (APACHE) II severity of illness score could not be determined and no comment can be made on the appropriateness of antimicrobial chemotherapy, since the initial reason for therapy, dosage, the administration route and the duration of treatment were unknown. However, it is known that the majority of patients were exposed to two or more antimicrobial agents during hospitalisation, such as carbapenems and glycopeptides. This is worrying, due to the strong association between the over usage of antimicrobials and the selection of resistance in microorganisms. The device utilisation rate could not be calculated, since the number of in-patient days, for the wards where catheter days were collected, was unknown. Lastly, the study cannot be extrapolated to the entirety of South Africa, since a single institution is not representative of the country.

## Conclusions

The microbiology laboratory can obtain baseline data on the CRI rates in a setting with limited resources by following the catheter culture requests submitted to the laboratory. The occurrence of MC-CRBSI can be decreased by implementing and adhering to central line insertion and maintenance bundles, strictly following hand hygiene procedures, as well as environmental decontamination, not only to prevent CRIs, but to limit the spread of multidrug-resistant bacteria.

## Abbreviations

APACHE II: Acute Physiological Assessment and Chronic Health Evaluation II
BCA: Best Care…Always!
BC(s): Blood culture(s)
CRIs: Catheter-related infections
CLABSI: Central line-associated bloodstream infection
CDC/NHSN: Centres of Disease Control and Prevention’s National Healthcare Safety Network
cfu: colony forming units
ICU: Intensive care unit
MC-CRBSI: Microbiologically confirmed catheter-related bloodstream infection
MDR: Multidrug-resistance
PDR: Pandrug-resistance
SD: Standard deviation
XDR: Extensively drug-resistance
